# Clinical implications of Girdin and PI3K protein expression in breast cancer

**DOI:** 10.3892/ol.2013.1249

**Published:** 2013-03-12

**Authors:** FENG JIN, CAIGANG LIU, YANG GUO, HAO CHEN, YUNFEI WU

**Affiliations:** Department of Breast Surgery, General Surgery, The First Hospital of China Medical University, Shenyang 110001, P.R. China

**Keywords:** breast cancer, Girdin, PI3K, stem cell, survival

## Abstract

The aim of this study was to investigate the correlation between Girdin and PI3K in breast cancer stem cells and the clinical implications of the co-expression of these two proteins in breast cancer patients. CD44^+^/CD24^−^ tumor cells from the MD-231 cell line were sorted by flow cytometry. The expression status of Girdin and PI3K proteins was detected using western blotting and immunohistochemical staining. The relationship between Girdin and PI3K proteins and clinicopathological parameters was analyzed in 820 breast cancer patients. Girdin and PI3K proteins were more highly expressed in CD44^+^/CD24^−^ tumor stem cells compared to the control group and Girdin and PI3K proteins were co-immunoprecipitated in the MD-231 cell line. Of the 820 enrolled breast cancer patients, Girdin and PI3K proteins were expressed in 295 (35.98%) and 492 (60.00%) cases, respectively. There were 162 (19.76%) cases which co-expressed Girdin and PI3K proteins. Univariate and multivariate analyses indicated that the co-expression of Girdin and PI3K proteins correlated with histological type, metastatic nodes and distant metastasis (P=0.01, 0.001 and 0.001, respectively). After analyzing survival rates, cases with Girdin and PI3K co-expression were shown to attain a significantly increased distant metastasis rate and poorer postoperative, disease-specific survival compared to those with Girdin and PI3K co-expression (P=0.001). In the Cox regression test, Girdin and PI3K co-expression was detected as an independent prognostic factor (P=0.001). Girdin may regulate the biological behavior of breast cancer via the PI3K/Akt/mTOR pathway, and thus, serve as a potential new target for breast cancer treatment.

## Introduction

In human epithelial cancers, the PI3K-Akt signaling pathway is frequently hyperactivated during cancer invasion, and the progressive enhancement of PI3K-Akt coupled to efficient cell migration is a hallmark of high metastatic potential (1–3). Although many identified molecules have a role in breast cancer progression and metastasis, the mechanisms involved are far from clear (4,5). To date, few molecules exhibit a high efficiency in predicting postoperative distant metastasis for breast cancer.

In our recent study, Girdin was highly expressed in breast cancer and was a potential biomarker for the initiation, progression and differentiation of breast cancer tumors (6). Another study observed that the expression of Girdin associated with PI3K-Akt signaling, actin remodeling, motility and invasion varies among tumors, and the majority of them have failed to make a transition into cancer clinics as prognostic biomarkers (7). Girdin is a novel protein, found at the crossroads of G protein signaling and tyrosine kinase receptor signaling (8). When the epidermal growth factor receptor signal is activated, Girdin is directly activated by Akt (9). Recently, Ghosh *et al* discovered a Girdin-Gαi molecular complex that binds to the epidermal growth factor receptor and determines whether cells migrate or proliferate (9). The authors also suggested that the expression of Girdin predicts patient survival in colon cancer and may serve as a useful adjunct to traditional staging strategies in colorectal carcinoma (9).

Currently, studies addressing the function and specific mechanism of Girdin and the PI3K-Akt signaling pathway in regulating the biological behavior of breast cancer remain rare. Moreover, the correlation between the expression status of Girdin and PI3K proteins remains unclear. In this study, we investigated the expression status of Girdin and PI3K proteins and the clinical implications in the management of breast cancer.

## Materials and methods

### Patients and tissue specimens

In this study, we selected 820 patients who had histologically confirmed breast cancer and had undergone radical surgery in the Liaoning Province Tumor Hospital between January 2003 and December 2006. The inclusion criteria were: a) curative surgery had been performed; b) resected specimens had been pathologically examined; c) >15 lymph nodes had been pathologically examined after surgery; and d) a complete medical record was available. Of 820 enrolled patients, 591 had received adjuvant chemotherapy and 165 had received adjuvant radiotherapy. Of the 591 cases that had received adjuvant chemotherapy, 153 developed distant postoperative metastasis. Additionally, 52 of the 165 patients who received adjuvant radiotherapy also developed postoperative metastasis. This study was approved by the Ethics Committee of China Medical University, Shenyang, China.

### Experiment materials

Polyclonal rabbit antihuman Girdin and PI3K antibodies (dilution 1:100) were purchased from Santa Cruz Biotechnology (Santa Cruz, CA, USA). The monoclonal mouse antihuman ER, PR and anti-CerbB2 antibodies, all with a dilution factor of 1:100, were purchased from Dako (Carpinteria, CA, USA). CD24-PE and CD44-FITC were purchased from BD Pharmingen (San Diego, CA, USA) and the flow cytometer used in this study was a FACSVantage obtained from BD Pharmingen.

### Flow cytometry test

Cells were counted and transferred to a 5-ml tube, washed twice with Hank’s balanced salt solution (HBSS) with 2% heat-inactivated calf serum (HICS; 5 min at 1,000 rpm), then resuspended in 100 *μ*l (per 10^6^ cells) of HBSS/2% HICS (10). Then, 5 *μ*l of Sandoglobin solution (1 mg/ml) was added and incubated on ice for 10 min, after which the sample was washed twice with HBSS/2% HICS and resuspended in 100 *μ*l (per 10^6^ cells) of HBSS/2% HICS. Antibodies (appropriate dilution per antibody) were added and incubated for 20 min on ice, and then washed twice with HBSS/2% HICS and flow cytometry was performed. Cells were routinely sorted twice, and then reanalyzed for purity, typically >95%. Dead cells were removed using the viability dye 7AAD. CD44^+^/CD24^−^ tumor cells were selected by CD44 and CD24.

### Western blot analysis

For western blot analysis, cells were lysed with the buffer [0.1% SDS, 50 mmol/l Tris-HCl (pH 7.6), 1% NP-40, 150 mmol/l NaCl, 2 mg/ml aprotinin, 2 mg/ml leupeptin and 7 mg/ml PMSF] (10). Protein concentrations were determined using a BCA Protein Assay kit (Pierce Biotechnology, Inc., Rockford, IL, USA). Proteins (30 *μ*g) were separated on 10% SDS-PAGE gels and transferred to a PVDF membrane. After blocking, the membrane was incubated with the primary antibody (1:500, Biorbyt Ltd., Cambridge, UK) at 4°C overnight. After washing, the membrane was incubated with a secondary antibody at a dilution of 1:2,000 at room temperature for 1 h. Proteins were detected using an ECL kit (Varsal Instruments, Beijing, China), and anti-β-actin antibody (Sigma-Aldrich, St. Louis, MO, USA) was used as a loading control. Densitometry was performed using Gel-pro Analyzer software (Media Cybernetics, Silver Spring, MD, USA).

### Immunohistochemistry experimental procedures

We fixed thin slices of tumor tissue from all the cases we received in our histopathology unit in 4% formaldehyde solution (pH 7.0) for <24 h (11). The tissues were processed routinely for paraffin embedding, and 4-*μ*m-thick sections were cut and placed on glass slides coated with (3-aminopropyl)triethoxysilane for immunohistochemistry. Tissue samples were stained with hematoxylin and eosin to determine histological type and tumor grade.

Breast tumor tissues and non-neoplastic breast tissues were cut at a thickness of 4 *μ*m using a cryostat. The sections were mounted on microscope slides, air-dried and fixed in a mixture of 50% acetone and 50% methanol. The sections were de-waxed with xylene, gradually hydrated with gradient alcohol and washed with phosphate-buffered saline (PBS). Sections were then incubated for 60 min with the primary antibody. Following washing with PBS, the sections were incubated for 30 min in the secondary biotinylated antibody (multilink swine anti-goat/mouse/rabbit immunoglobulin; Dako). Following washing, avidin-biotin complex (1:1,000 dilution; Vector Laboratories, Burlingame, CA, USA) was applied to the sections for 30–60 min at room temperature. The immunoreactive products were visualized by the catalysis of 3,3′-diaminobenzidine (DAB) using horseradish peroxidase in the presence of H_2_O_2_ following extensive washing. Sections were counterstained in Gill’s hematoxylin and dehydrated in ascending grades of methanol prior to being cleared in xylene and mounted under a coverslip.

Girdin and PI3K expression was classified semi-quantitatively according to the following criteria: 0 if <1% of the neoplastic cells discretely expressed Girdin in their cytoplasm; 1+ if ≥1 but <10% of morphologically unequivocal neoplastic cells discretely expressed Girdin in their cytoplasm; and 2+ if ≥10% of morphologically unequivocal neoplastic cells discretely expressed Girdin in their cytoplasm. We considered samples scored as either 1+ or 2+ as positive.

### Statistical analysis

All data were analyzed using SPSS statistical software (Version 13.0, Chicago, IL, USA). Correlations between tumor markers and other parameters were studied using the Chi-square test and Fisher’s test or an independent t-test. Disease-specific survival was analyzed using the Kaplan-Meier method. The log-rank test was used to analyze differences in survival. Multivariate analysis was performed using the Cox proportional hazards model selected in forward stepwise. P<0.05 was considered to be statistically significant.

## Results

### Girdin and PI3K protein expression status in breast cancer stem cells

**Figure 1:**
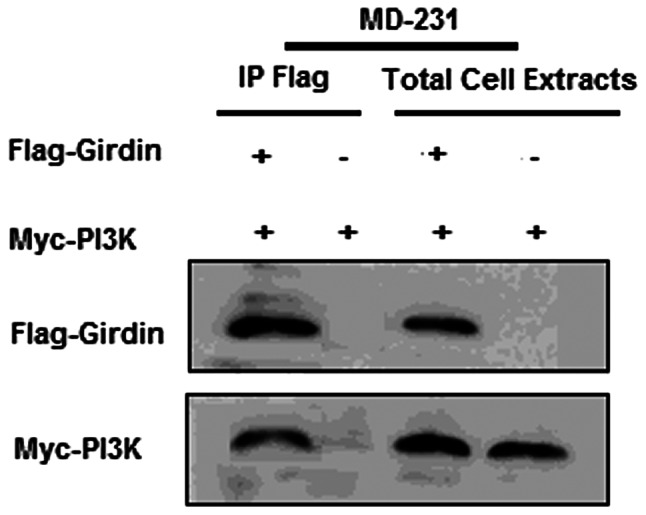
Girdin and PI3K protein co-immunoprecipitation in the MD-231 cell line.

The CD44^+^/CD24^−^ tumor cells from the MD-231 cell line were sorted by flow cytometry. After seven days of serum-free suspension culture, single-cell suspensions of cancer stem cells that were separated from the solid tumors produced viable mammospheres (20–100 *μ*m). After western blot analysis, Girdin and PI3K proteins were expressed at a higher level in cancer stem cells compared to the control cells (Fig. 1A). Furthermore, Girdin and PI3K protein co-immuno-precipitation was observed (Fig. 1B).

### Patient characteristics

Of the 820 enrolled breast cancer patients, Girdin and PI3K proteins were expressed positively in 295 (35.98%) and 492 (60%) cases, respectively. In 162 (19.76%) cases, Girdin and PI3K proteins were co-expressed (Table I). After univariate analysis, the co-expression of Girdin and PI3K protein was correlated with histological type, metastatic nodes and distant metastasis (P=0.001, 0.001 and 0.001, respectively). In order to exclude the influence of confounding factors, we compared the age, tumor size, histological grade and adjuvant treatment between the patients with distant metastasis and those without. No difference was observed between the patients with distant metastasis and those without.

### Correlation between Girdin and PI3K protein co-expression and clinicopathological characteristics

**Figure 2:**
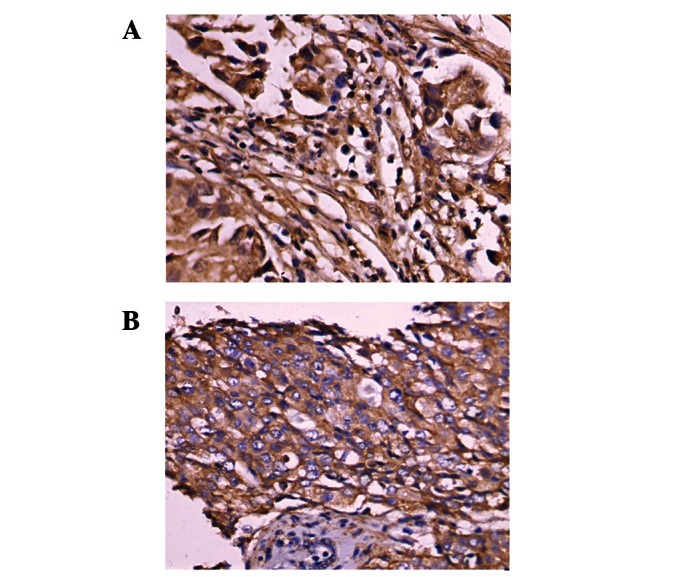
Immunohistochemical examination showed Girdin and PI3K proteins were located (A) at the cytoplasm and membrane and (B) cytoplasm of breast cancer cells. SP staining. Magnification, ×400.

Immunohistochemical examination demonstrated that Girdin and PI3K proteins were located at the cytoplasm and membrane of breast cancer cells (Fig. 2). Moreover, we observed that Girdin and PI3K protein co-expression was related to histological type, meta-static nodes and distant metastasis (P=0.001, 0.001, and 0.001, respectively). Following multivariate analysis, Girdin and PI3K protein co-expression was shown to be related to histological type, metastatic nodes and distant metastasis (P=0.01, 0.001 and 0.001, respectively) (Table II).

### Prognostic analysis

**Figure 3:**
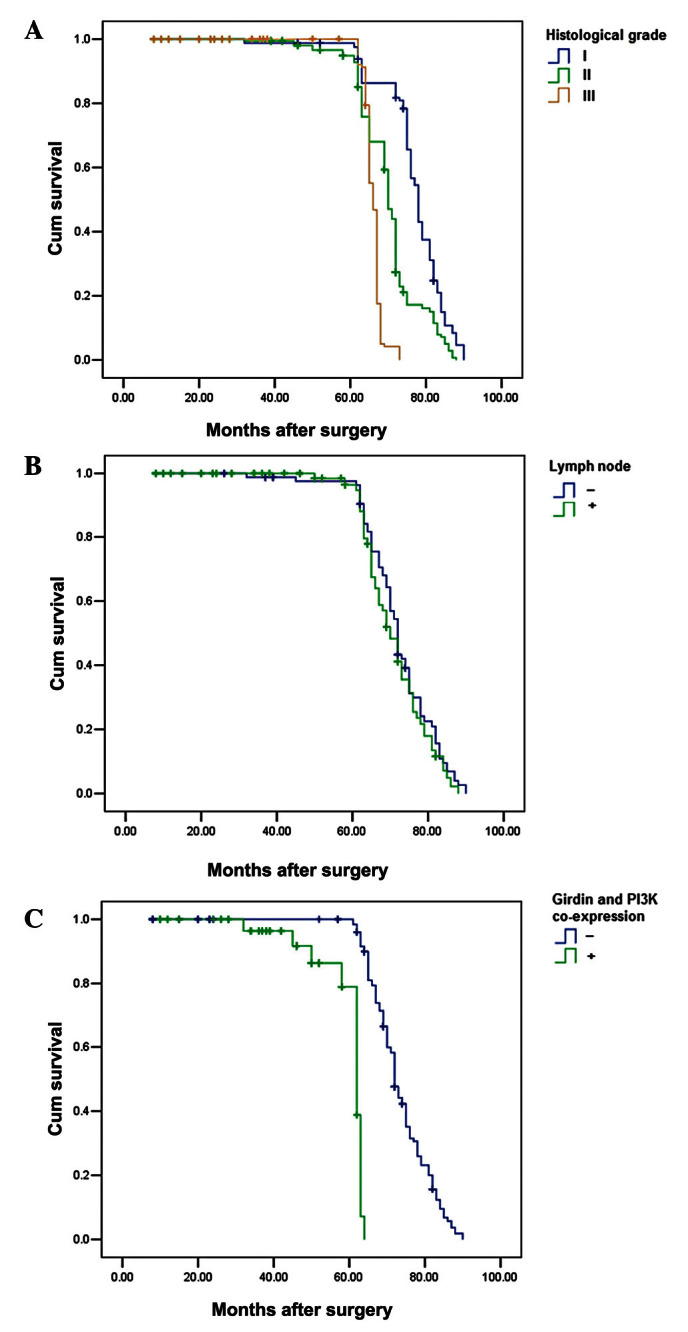
(A and B) After analyzing survival rates, histological grade and lymph node metastasis were also significantly correlated with postoperative survival (P=0.01 and 0.001). (C) Co-expression of Girdin and PI3K was associated with breast cancer-specific survival in all 820 cases (P= 0.001, log-rank test).

After analyzing survival rates, cases with Girdin and PI3K co-expression were shown to have a significantly increased distant metastasis rate and poorer postoperative, disease-specific survival than those with Girdin and PI3K co-expression (P=0.001) (Fig 3). In addition, histological grade and lymph node metastasis were also significantly correlated with the postoperative survival (P=0.01 and 0.001) (Fig. 3). In the Cox regression test, Girdin and PI3K co-expression was detected as an independent prognostic factor (P=0.001; Table III).

## Discussion

Currently, the expression status of Girdin protein in breast cancer and its correlation with the biological behavior of breast cancer remains unclear (12). Furthermore, few studies have addressed Girdin expression in breast cancer and its correlation with the prognosis of breast cancer (13).

In our recent study, the Girdin protein was found to be related to histological grade and distant metastasis of breast cancer, and Girdin expression was significantly related to both CerbB2 and Ki67 expression (6). Dunkel *et al* reported that GIV is a metastasis-related protein, serving as both a therapeutic target and a prognostic biomarker in cancer patients, and Girdin is a direct target of the transcription factor signal transducer and activator of transcription-3 (STAT3) (14). In another study, Girdin was considered to be a stem cell gene related to the histological grade and distant metastasis of colorectal cancer. Cases with high Girdin protein expression levels were shown to attain a significantly higher rate of liver metastasis and poorer postoperative, disease-specific survival than those with no or low levels of Girdin protein expression in colorectal cancer (15).

In this study, it was observed that Girdin and PI3K proteins were highly expressed in breast cancer tumor stem cells and Girdin and PI3K proteins co-immunoprecipitated in the MD-231 cell line. The results indicated that Girdin may have an important role in breast cancer stem cells. We further investigated the correlation between Girdin and PI3K co-expression and the biological behavior of breast cancer. Finally, co-expression of Girdin and PI3K protein was related to histological type, metastatic nodes and distant metastasis and the cases with Girdin and PI3K co-expression attained a poor postoperative, disease-specific survival. In the Cox regression test, Girdin and PI3K co-expression was detected to be an independent prognostic factor.

In a recent study, Natsume *et al* discovered that Girdin is highly expressed in human glioblastoma. Stable Girdin knockdown in isolated glioblastoma stem cells resulted in the decreased expression of stem cell markers, including CD133, induced multilineage neural differentiation, and inhibited *in vitro* cell motility, *ex vivo* invasion, the capacity for sphere-formation and *in vivo* tumor formation (16). The outcome of the study indicated that Girdin is required for glioblastoma-initiating stem cells to sustain their stemness and invasive properties. Girdin has been reported as a multidomain signaling molecule that enhances PI3K-Akt signals downstream of both G protein-coupled and growth factor receptors (17). However, there has been no study based on the correlation between Girdin and PI3K in breast cancer, although it was reported as a novel substrate of Akt. The outcomes of this study illustrated that Girdin and PI3K have a linear correlation in breast cancer and they may be necessary to the PI3K/Akt/mTOR pathway. However, the specific mechanism involved requires further investigation.

## Figures and Tables

**Table I:** Correlations between Girdin and PI3K co-expression and clinicopathological features.

Variables	n	Girdin^+^/PI3K^+^ [n(%)]	χ^2^ value	P-value
Age(years)			0.135	0.713
<35	124	26 (20.97)		
>35	696	136 (19.54)		
Tumor size			1.778	0.409
T1	167	27 (16.17)		
T2	509	104 (20.43)		
T3	144	31 (14.58)		
Histological grade			62.002	0.001
I	104	11 (10.58)		
II	520	94 (18.08)		
III	196	57 (29.08)		
Metastatic nodes			11.656	0.001
Negative	392	58 (14.80)		
Positive	428	104 (24.30)		
Distant metastasis			40.863	0.001
Negative	599	86 (14.36)		
Positive	221	76 (34.39)		
Triple-negative breast cancer			0.257	0.612
Yes	131	28 (21.37)		
No	689	134 (19.45)		

**Table II:** Multivariate analysis of the factors related to Girdin and PI3K co-expression.

Characteristic	Exp (B)	95% CI for Exp (B)	P-value
Age	0.528	0.378–1.408	0.270
Tumor size	1.074	0.542–1.860	0.164
Histological type	2.612	1.264–4.105	0.01
Metastatic node	3.765	1.059–2.114	0.001
Distant metastasis	4.156	1.958–6.426	0.001
Triple-negative breast cancer	1.285	0.836–1.610	0.082
Constant	0.032		

CI, confidence interval.

**Table III:** Cox model regression analysis of the colorectal cancer prognostic factors.

Variables	OR	95% CI for OR	P-value
Age	1.182	0.725–1.884	0.261
Tumor size	1.403	0.654–2.071	0.154
Histological type	1.628	1.319–3.166	0.020
Metastatic node	2.135	1.508–3.967	0.010
Triple-negative breast cancer	2.753	1.472–4.324	0.001
Girdin and PI3K co-expression	3.420	1.563–5.182	0.001

OR, odds ratio; CI, confidence interval.
